# M. Metchnikoff on the Future of Man

**Published:** 1903-08-08

**Authors:** 


### M. Metchnikoff on the Future of Man.

It is always instructive to hear what a specialist has to say upon some great question wider than his speciality. He may be a thinker, and his thinking will be all the better for his trained habits of observation and reasoning in one small corner of the field. M. Elie Metchnikoff, who began his career a good many years ago as a zoologist, is best known for his work as a bacteriologist at the Pasteur Institute in Paris, and more especially for his comparatively recent book on Immunity. As a relaxation from the microscope and the cultivation-dish, he has just produced a philosophical essay (Paris 1903) under the comprehensive title of "Studies of Human Nature: an Essay in Optimistic Philosophy." He believes, or he has persuaded himself, that the defects of our nature are remediable, if we will follow the teachings of science, and that the future of man may become progressively happier. Religion and the moral law do not come within the scope of his studies at all, so far not so that he would appear to be looking for the means of human perfectibility without those ancient aids. This is a point of view that has been taken by certain writers ever since the full bearings of Darwinism began to be discussed, and, as it is not usual, so it need not now excite prejudice to the extent of denying the author a hearing. But, prejudice apart, we do not find that M. Metchnikoff is profound in his treatment of the matter. His optimism appears to have arisen in great part from his confidence in the future achievement of bacteriology, or microbiology, or the healing art as a whole. Even old age, he thinks, is a malady which is remediable within limits. It is a commonplace of pathology that certain tissues, especially the walls of the arteries, become hard and stiff in old age, and that they may become prematurely so under various influences. One of those influences is his original discovery—the toxins which are produced in the great intestine by microbes and are absorbed into the circulation, producing sclerosis of the tissues. Much may be done, he thinks, to remove such morbid influences tending to shorten life; so that we shall live to the age of the patriarchs, enjoying a large measure of vigour and benefiting the community by the wisdom of age.

In these matters of disease and old age, the optimist philosopher of human nature is either a sanguine bacteriologist or a moralist in disguise. But in another section he is on strictly revolutional ground. This is the question of man's physical imperfections, due to his descent from lower forms. It is now admitted that we are not so truly the paragon of animals as might be wished, at least in points of structure and function. Helmholtz has shown that the eye is far from being a perfect optical instrument. We bear in our bodies many traces of imperfect adaptation to our environment, as well as some apparently useless survivals from ancestral types. All such structural imperfections, together with defective and aberrant instincts, and whatever else may be included under the old phrase, "the more obvious defects of our animality," may be corrected upon scientific lines by such means as selection (otherwise called breeding), and by a more exact adaptation between the organism and its surroundings. What we miss most in this hopeful view of man's future is any notice of the principle of Reversion, or slipping back. It was in Darwin's second great work, on "Domestication," that he said most about reversion, perhaps because we see most of it under artificial conditions of living. Tennyson, who followed all these scientific ideas closely, appears to have been more impressed by Reversion than even Darwin himself. While Evolution has made M. Metchnikoff an optimist, Reversion made Tennyson a pessimist—or, at all events, as much of a pessimist as the author of the New Locksley Hall was:

> "Evolution ever climbing after some ideal good,
> And Reversion ever dragging Evolution in the mud."

It is hard to say how much the writer of that succinct couplet may not have intended to include in his thought. But if he had been a pathologist he could hardly have expressed more correctly the principle which underlies a large part of the diseases of modern life. M. Metchnikoff is a pathologist who has essayed to be an evolutional philosopher, but he has failed to do justice to the reversion side of the problem. If he had done so, he might have been converted from his optimism.

